# Measurement Tools for Radiation‐Induced Fibrosis in Head and Neck Cancer: A Systematic Review

**DOI:** 10.1002/hed.70380

**Published:** 2026-07-02

**Authors:** Vanessa Helou, Pooja D. Reddy, Purva H. Rumde, Mary L. Klem, Sagar G. Kansara, Oldrich Virag, Yvonne M. Mowery, Nicole N. Scheff, Matthew E. Spector, Shaum Sridharan, Marci L. Nilsen, Kevin J. Contrera

**Affiliations:** ^1^ Department of Otolaryngology University of Pittsburgh School of Medicine Pittsburgh Pennsylvania USA; ^2^ University of Pittsburgh School of Medicine Pittsburgh Pennsylvania USA; ^3^ Health Sciences Library System University of Pittsburgh Pennsylvania USA; ^4^ Department of Otolaryngology Louisiana State University School of Medicine New Orleans Louisiana USA; ^5^ Department of Radiation Oncology UPMC Hillman Cancer Center Pennsylvania USA; ^6^ Department of Neurobiology University of Pittsburgh Pittsburgh Pennsylvania USA

**Keywords:** fibrosis measurement, head and neck cancer, radiation‐induced fibrosis, systematic review

## Abstract

**Background:**

Radiation‐induced fibrosis is a common, often debilitating late effect of head and neck cancer (HNC) treatment. An updated systematic review is needed to evaluate the reliability, validity, and clinical utility of fibrosis measurement tools.

**Methods:**

Cochrane Library, Medline, Embase.com, and Web of Science were systematically searched. Articles were independently screened by two reviewers using COSMIN checklists for risk of bias assessment, following PRISMA guidelines.

**Results:**

Of 4678 articles, 17 studies describing 13 tools met inclusion: six studies assessed four patient‐reported outcome measures, two assessed two clinician‐reported tools, and nine assessed seven imaging modalities. The Neck Fibrosis Scale, HN‐LEF Symptom Inventory, and LENT‐SOMA demonstrated validity and reliability. Young's modulus was the only imaging tool demonstrating both. Imaging studies had a higher risk of bias.

**Conclusion:**

Several subjective tools are reliable and valid for radiation‐induced fibrosis assessment in HNC. Young's modulus is the only objective imaging‐based tool that provides evidence of both validity and reliability, though methodological limitations remain.

## 1 Introduction

The primary treatment modalities for head and neck cancers (HNC) remain surgery and radiation therapy (RT). Approximately 60% of patients with locally advanced disease are treated with a combination of these modalities [1]. RT induces DNA damage in cancer cells, leading to cell death primarily through apoptosis [2]. However, this damage is not exclusive to malignant cells, as surrounding healthy tissue is also affected despite advances in RT precision [3, 4]. This results in a spectrum of long‐term complications.

Radiation‐induced fibrosis (RIF) is a common late complication that may develop months to years following RT [5]. The prevalence of RIF varies widely depending on the definition, assessment method, and time point. The cumulative incidence of severe subcutaneous fibrosis has been reported to exceed 40% at 10 years in patients with prior neck dissection or high radiation doses [6], and RIF has been reported in up to 50% of patients with head and neck squamous cell carcinoma following RT [7]. Importantly, RIF is not a static condition: both its prevalence and severity may continue to increase for many years following treatment [8]. The pathophysiology of RIF involves multiple interconnected mechanisms, including RT‐induced inflammation that promotes fibroblast differentiation into myofibroblasts [9, 10]. These cells drive tissue remodeling through excessive collagen deposition and extracellular matrix production. In parallel, cellular senescence resulting from oxidative stress and DNA damage leads to a senescence‐associated secretory phenotype characterized by increased mitogenic and immunomodulatory cytokines, accounting for the chronic and progressive nature of injury [9]. The resultant fibrosis reduces tissue compliance, which leads to functional and cosmetic impairments. Over time, RIF‐related complications significantly impair quality of life, underscoring the need for reliable and valid tools to assess fibrosis following RT and throughout survivorship [7, 11].

Although numerous objective and subjective modalities have been proposed to assess RIF, there remains no consensus on a valid, reliable, and non‐invasive tool for patients with HNC [12]. Subjective approaches include patient‐reported outcome measures (PROMs) and clinician‐based palpation grading scales, whereas objective approaches encompass a range of imaging‐based techniques. The most recent systematic review of this topic was published by Shaw et al. in 2016 [12], highlighting a critical gap in the literature given the proliferation of assessment tools developed since that time. Although lymphedema and fibrosis frequently coexist in HNC survivors and are often discussed together, they represent distinct pathological processes requiring different assessment approaches. Lymphedema results from the pathological accumulation of interstitial fluid due to lymphatic system dysfunction and is assessed using volumetric tools and staging systems [13, 14]. Fibrosis, by contrast, is characterized by the overaccumulation of fibrotic tissue with associated loss of elasticity and tissue hardening, and is assessed using palpation‐based grading scales and mechanical or imaging‐based tools that measure tissue stiffness [6, 12]. A dedicated systematic review of lymphedema assessment in HNC has been published [14]. This review therefore focuses specifically on measurement tools for fibrosis. It should be noted, however, that some assessment tools evaluate both lymphedema and fibrosis simultaneously, and these are included where fibrosis assessment is a component [15, 16, 17]. Accordingly, the aim of this systematic review is to identify existing subjective and objective RIF measurement tools used in HNC and to evaluate their reported reliability and validity.

## 2 Material and Methods

This systematic review is being reported in accordance with the updated Preferred Reporting Items for Systematic Reviews and Meta‐Analyses (PRISMA) statement [18].

### 2.1 Search Strategy

A comprehensive literature search was performed across the following electronic databases: the Cochrane Library (Wiley), Embase (Elsevier), Ovid MEDLINE (Wolters Kluwer), and Web of Science (Clarivate). The MEDLINE search strategy was developed by a health sciences librarian (M.L.K.) using a combination of Medical Subject Headings (MeSH) and natural language terms to create two concept clusters: “radiation fibrosis” and “head and neck cancer.” Terms within each cluster were combined using the Boolean operator “OR”, and the final strategy combined the two clusters using the Boolean operator “AND.”

The MEDLINE strategy was adapted for the remaining databases using database‐specific controlled vocabulary when available. Searches were initially conducted on November 11, 2024, with an English‐language restriction applied. An updated search was performed on February 4, 2026. All records were imported into EndNote (version X9; Clarivate Analytics), and duplicates were removed using Deduklick [19]. The remaining unique records were uploaded to DistillerSR (version 2023.8.2; DistillerSR Inc., 2024) for screening [20].

### 2.2 Screening and Eligibility Criteria

Two independent reviewers (P.D.R. and P.H.R.) screened titles and abstracts for relevance to RIF measurement tools. Full‐text screening was subsequently performed using predefined eligibility criteria. Disagreements were resolved through discussion; when consensus could not be reached, a third reviewer (K.J.C and V.H.) adjudicated. The study selection process is summarized in the PRISMA flow diagram (Figure 1).

**FIGURE 1:**
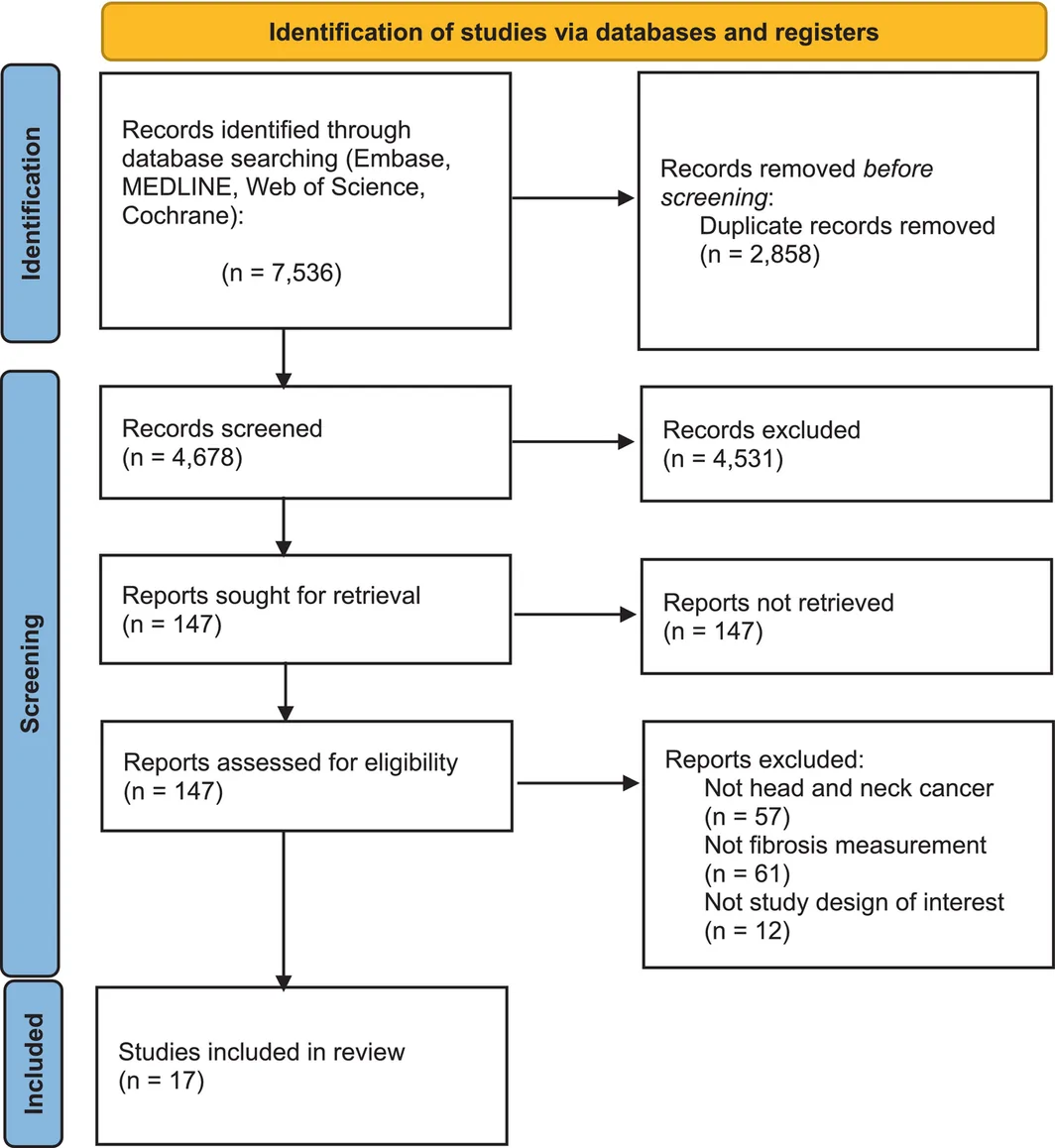
PRISMA 2020 flow diagram of study selection process. [Color figure can be viewed at wileyonlinelibrary.com]

Studies were included if they:

1. Involved adult patients (≥ 18 years) with a diagnosis of HNC treated with RT for curative or palliative intent in whom post‐treatment fibrosis or fibrosis‐related outcomes were evaluated.
2. Evaluated subjective and/or objective measurement tools for RIF.
3. Were original research articles, including randomized controlled trials, quasi‐randomized trials, cohort studies, case–control studies, cross‐sectional studies, clinical trials, unpublished or non‐peer‐reviewed sources including conference abstracts, dissertations, and theses.

Studies were excluded if they (1) involved non‐human participants; (2) were abstracts, case reports, case series, editorials, or review articles; or (3) were published in languages other than English.

### 2.3 Data Extraction and Synthesis

Data extracted from each included study included authorship, year of publication, study design and duration, cancer site, participant demographics, radiation treatment details, time since treatment completion, fibrosis measurement tool(s), tool type, fibrosis type (classified as internal, external, or both, based on the anatomical structures assessed), assessments of validity and reliability, and key outcomes. Data were recorded using a standardized extraction form and synthesized descriptively, with results summarized in tables. Summary statistics, including medians, interquartile ranges (IQR), and ranges, were calculated. Due to the heterogeneity of measurement tools and outcome definitions, quantitative meta‐analysis was not performed.

### 2.4 Risk of Bias Assessment

Risk of bias was assessed using the COnsensus‐based Standards for the selection of health Measurement INstruments (COSMIN) Risk of Bias checklist [21]. This tool evaluates methodological quality across 10 domains, including instrument development, content validity, structural validity, internal consistency, cross‐cultural validity, reliability, measurement error, criterion validity, hypothesis testing for construct validity, and responsiveness. Discrepancies were resolved by consensus.

## 3 Results

Of 4678 records screened, 17 studies met the inclusion criteria, describing 13 distinct measurement tools for RIF in HNC patients. Six studies (35.3%) evaluated four PROMs, two (11.8%) evaluated two clinician‐reported outcome measures, and nine (52.9%) evaluated seven imaging‐based tools.

The median year of publication was 2021 (IQR, 2009–2025). The median study sample size was 50 (IQR, 30–105; range, 12–220). The majority of studies were conducted in single‐center settings. Cancer sites included nasopharyngeal carcinoma, oral cavity, oropharyngeal, laryngeal, and hypopharyngeal cancers, with many studies including mixed HNC populations without detailed site‐specific breakdown. Fibrosis assessment locations included the cervical soft tissues (most common), sternocleidomastoid muscle, submental region, submandibular region, and pharyngeal structures [15, 22]. The neck was the predominant site of assessment across all measurement modalities.

When classified by fibrosis type, 12 of the 17 studies (70.6%) assessed external fibrosis only, targeting the skin, subcutaneous tissues, and superficial cervical musculature. Five studies (29.4%) assessed both external and internal fibrosis (Table S1). Among PROM studies, only those using instruments designed to capture both lymphedema and fibrosis symptoms (Head and Neck External Lymphedema and Fibrosis (HN‐LEF) Symptom Inventory, Dutch LSIDS‐H&N v2.0) addressed internal fibrosis [17, 23, 24], while studies using the fibrosis‐specific neck fibrosis scale (NFS) and the LENT‐SOMA Patient Questionnaire assessed external fibrosis only [25, 26, 27]. Treatment modalities include RT alone, surgery with adjuvant RT, and concurrent chemoradiation. Notably, the majority of studies (13, 76%) did not report radiation dose parameters. One study reported a median dose of 50 Gy (range, 40–70 Gy) with a mean dose of 51.1 Gy [27]. Another study reported a mean sternocleidomastoid muscle absorbed dose of 54.5 ± 7.1 Gy [28]. One study reported biological effective doses of 110–116.9 Gy_3_ to the upper‐middle neck with an additional 30–33.3 Gy_3_ boost [29]. Additionally, one study reported mean right and left neck doses stratified by symptom status, with mean doses ranging from approximately 59–61 Gy [30]. Reporting of fractionation was similarly limited. One study reported a median of 16 fractions (range, 15–35) [27], while another reported 30 fractions in 80% of patients and 35 fractions in 20% [28]. The remaining studies did not report fractionation schedules. The detailed study characteristics are summarized in Tables 1 and S1.

**TABLE 1:** Patient‐reported, clinician‐reported, and imaging‐based tools for measuring radiation‐induced fibrosis in head and neck cancer: Study characteristics, evidence of validity, reliability, and risk of bias.

RIF measurement type	Measurement tool	Publication	Sample size^a^	Clinical evidence of validity	Clinical evidence of reliability^b^	Risk of bias^c^
PROMs	Neck fibrosis scale	Noel 2022 [25]	94	Factor analysis during item reduction; face and content validity via expert and patient input	Test–retest ICC = 0.95; Cronbach's *α* = 0.95	Adequate
		Saleem 2023 [26]	129	Confirmed 2‐factor structure; Physical domain correlated with neck elasticity (*ρ* = 0.902, *p* < 0.01)	Physical domain: ICC = 0.85; Emotional domain: ICC = 0.48; Cronbach's *α* > 0.8 for each domain	Adequate
	HN‐LEF Symptom Inventory	Deng 2021 [17]	117	Content validity with patient and expert input; 7 symptom domain clusters identified	Cronbach's *α* > 0.8 across domains	Adequate
		Deng 2022 [23]	117	Construct validity confirmed with EORTC QLQ‐C30, NDI, SF‐MPQ	Not formally assessed	Adequate
	LENT‐SOMA Patient Questionnaire	Ho 2010 [27]	220	Face/content validity; Construct validity via correlation with EORTC QLQ‐C30/H (*ρ* > 0.6, *p* < 0.001)	Cronbach's *α* = 0.786	Adequate
	Dutch LSIDS‐H&N v2.0	Van Aperen 2026 [24]	30	Strong convergent validity (10/11 hypotheses accepted)	Test–retest ICC = 0.792–0.963; Cronbach's *α* = 0.883–0.981	Adequate
Clinician‐reported	HN‐LEF assessment criteria	Deng 2016 [15]	51	Concurrent validity with ultrasound examination findings	Interrater: 91.0% agreement (Kappa = 0.81); Intrarater: 96.1% agreement	Adequate
	Courage Kenny‐Fibrosis grading scale	Gilchrist 2025 [31]	15 head and neck cancer survivors (of 41 total cancer survivors)	Face validity (iterative survey with 43 clinicians); Concurrent validity with CTCAE, LENT‐SOMA (rS = 0.290–0.574, *p* < 0.05)	Inter‐rater ICC = 0.810; Intra‐rater ICC = 0.941	Adequate
Imaging—ultrasound	Young's modulus (ultrasound indentation)	Leung 2002 [29]	105	Significant positive correlation with hand palpation scores; Significant negative correlation with neck rotation range; Discriminates regions with differential radiation doses	Inter‐observer variation ±15.2%; Intra‐observer variation ±7.2% (95% CI)	Inadequate
		Zheng 2000 [32]	4 patients with post‐RT neck fibrosis, 8 controls	Feasibility study; Discriminated normal (12.8 ± 3.9 kPa) from fibrotic tissue (46.4–108.3 kPa)	Site‐dependence ±14.2%; Inter‐observer ±15.2%; Intra‐observer ±7.2%	Inadequate
		Huang 2005 [33]	105	Viscoelasticity parameters correlated with clinical fibrosis parameters and neck rotation range	Parameters discriminated tissue with different degrees of clinical fibrosis	Inadequate
	Shear wave elastography	Liu 2015 [22]	Reliability cohort: 30 (16 healthy, 14 NPC) Case–control cohort: 50 (25 NPC, 25 controls)	Significantly higher stiffness in post‐irradiated patients (64.6 ± 46.8 kPa) vs. controls (19.9 ± 7.8 kPa, *p* < 0.001); Correlated with muscle atrophy and subcutaneous tissue thinning	Intraoperator ICC = 0.90–0.99; Interoperator ICC = 0.77–0.94	Doubtful
	2D‐Shear wave elastography (Young modulus) B‐mode sequential image processing (Young modulus and shear modulus)	Khodadadi 2025 [28]	20 sternocleidomastoid muscles; patient number NR	Shear wave elastography: The Young's modulus derived from SWE did not detect radiation‐induced changes during radiotherapy (*p* = 0.611). B‐mode sequential image processing successfully detected early fibrosis. At 48 Gy: Young's modulus showed 100% specificity, 85% sensitivity, 93% accuracy; Shear modulus showed 95% specificity, 75% sensitivity, 85% accuracy. Parameters demonstrated significant dose‐dependent increase (*p* = 0.001). Limited validity at 3 months post‐RT: sensitivity dropped to 56% (Young's modulus) and 63% (shear modulus).	Not formally assessed	Inadequate
	Ultrasonic Nakagami imaging	Yang 2015 [30]	28 post‐RT patients (11 asymptomatic, 17 symptomatic), 12 controls	Significant differences between control, asymptomatic, and symptomatic groups (*p <* 0.001); Shape parameter increased 74.1%, scaling parameter increased 83.5% in symptomatic vs. control	Not formally assessed (feasibility/pilot study)	Inadequate
	High‐frequency ultrasound	Huang 2007 [34]	38	Significantly different skin thickness (*p <* 0.004) and integrated attenuation (*p* = 0.04) between patients and controls	Not formally assessed	Inadequate
Imaging—MRI	Quantitative MRI Biomarkers	Mao 2025 [35]	16	T2 SI differentiated edematous states (A‐B and C higher than D and No‐LEF); MRI structural volumes correlated with 10‐point HN‐LEF score (*p* < 0.05)	Not formally assessed (post hoc analysis)	Doubtful
Imaging—CT	CT‐LEFAT	West 2025 [16]	26	ROC AUC = 0.60–0.70 (limited diagnostic accuracy)	Inter‐rater kappa = 0.04–0.36 (slight to fair); Intra‐observer kappa = 0.44 (fair to moderate)	Inadequate

Abbreviations: AUC, area under the curve; CI, confidence interval; COSMIN, COnsensus‐based Standards for the selection of health Measurement INstruments; CT, computed tomography; CT‐LEFAT, computed tomography lymphedema and fibrosis assessment tool; CTCAE, common terminology criteria for adverse events; EORTC QLQ‐C30, European Organization for Research and Treatment of Cancer Quality of Life Questionnaire Core 30; EORTC QLQ‐H&N35, European Organization for Research and Treatment of Cancer Quality of Life Questionnaire Head and Neck Module 35; HNC, head and neck cancer; HN‐LEF, head and neck lymphedema and fibrosis; HN‐LEF‐SI, head and neck lymphedema and fibrosis symptom inventory; ICC, intraclass correlation coefficient; kPa, kilopascals; LENT‐SOMA, late effects normal tissue–subjective, objective, management, analytic; LSIDS‐H&N, lymphedema symptom intensity and distress survey–head and neck; MRI, magnetic resonance imaging; NDI, neck disability index; NFS, neck fibrosis scale; NR, not reported; PROMs, patient‐reported outcome measures; ROC, receiver operating characteristic; rS, Spearman correlation coefficient; RT, radiation therapy; SF‐MPQ, short‐form McGill pain questionnaire; SI, signal intensity; SWE, shear wave elastography; US, ultrasound; YM, Young's Modulus; *α*, Cronbach's alpha; *ρ*, Spearman's rho.

^a^ Sample sizes reflect the number of HNC patients included in the validation cohort. For studies including mixed cancer populations, the HNC‐specific sample size is reported in parentheses. For imaging studies, sample sizes may include both patients and healthy controls; these are reported separately where applicable.

^b^ Internal consistency was assessed using Cronbach's alpha (*α*), with values ≥ 0.70 considered acceptable and ≥ 0.80 considered good. Test–retest and inter−/intra‐rater reliability were assessed using intraclass correlation coefficients (ICC), with values ≥ 0.75 considered good and ≥ 0.90 considered excellent. For ultrasound indentation studies (Young's Modulus), reliability is reported as percentage variation rather than ICC.

^c^ Risk of Bias was assessed using the COSMIN Risk of Bias checklist. Ratings include: “Adequate” (low risk of bias with appropriate methodology), “Doubtful” (unclear methodology or minor concerns), and “Inadequate” (high risk of bias due to methodological flaws, small sample size, or incomplete assessment of measurement properties).

### 3.1 PROMs

Six studies evaluated four distinct PROMs for HNC fibrosis assessment. The characteristics and psychometric properties of each PROM are summarized below:

#### 3.1.1 NFS

The NFS was developed by Noel et al. as the first PROM designed exclusively to measure fibrosis symptoms in HNC patients [25]. Initial development involved four focus groups and expert opinion from a multidisciplinary group. The original 25‐item instrument was reduced to 15 items using the item impact method. Preliminary psychometric testing demonstrated excellent internal consistency (Cronbach's α = 0.95) and test–retest reliability (ICC = 0.95) [25].

Saleem et al. subsequently validated the domain structure of the NFS in 129 HNC patients with varying degrees of cutaneous neck fibrosis [26]. Exploratory factor analysis confirmed a two‐factor structure comprising “physical” and “emotional” domains, resulting in a revised 14‐item questionnaire. Internal consistency remained high (Cronbach's *α* > 0.8) for both domains. The physical domain demonstrated a strong correlation with neck elasticity (*ρ* = 0.902, 95% CI 0.839–0.972, *p* < 0.01), and patients receiving multimodal therapy had physical domain scores 31.6% higher (worse) than unimodal therapy patients [26].

#### 3.1.2 Head and Neck Lymphedema and Fibrosis Symptom Inventory (HN‐LEF‐SI)

Deng et al. refined and validated the HN‐LEF‐SI in 117 patients with oral cavity and oropharyngeal cancer assessed longitudinally from pretreatment to 12 months post‐treatment [17]. The original 64‐item LSIDS‐H&N was reduced to a 33‐item instrument following participant feedback that the original survey was burdensome. Seven symptom domain clusters were identified (including soft tissue and neurologic toxicity domains), all demonstrating good internal consistency (Cronbach's *α* > 0.8) [17]. Construct validity was subsequently confirmed through correlation with established measures including the EORTC QLQ‐C30, Neck Disability Index, and Short‐Form McGill Pain Questionnaire [17, 23].

#### 3.1.3 Late Effects Normal Tissue–Subjective, Objective, Management, Analytic (LENT‐SOMA) Patient Questionnaire

Ho et al. evaluated the LENT‐SOMA patient questionnaire for assessing late treatment toxicity in HNC patients post‐RT [27]. The instrument demonstrated adequate internal consistency (Cronbach's *α* = 0.79) and construct validity through significant correlation with EORTC QLQ‐C30 and QLQ‐H&N35 quality of life measures (*ρ* > 0.6, *p* < 0.001). Face and content validity were established through expert review [27].

#### 3.1.4 Dutch Lymphedema Symptom Intensity and Distress Survey‐Head and Neck (LSIDS‐H&N)

Van Aperen et al. performed cross‐cultural adaptation and validation of the Dutch version of the LSIDS‐H&N Version 2.0 [24]. The instrument demonstrated strong convergent validity, test–retest reliability (ICC = 0.792–0.963), and internal consistency (Cronbach's *α* = 0.883–0.981) [24].

### 3.2 Clinician‐Reported Outcome Measures

Two studies evaluated clinician‐reported outcome measures for HNC fibrosis assessment [15, 31].

#### 3.2.1 Head and Neck External Lymphedema and Fibrosis (HN‐LEF) Assessment Criteria

Deng et al. evaluated the reliability and validity of the HN‐LEF Assessment Criteria in 51 HNC patients who were at least 3 months post‐cancer therapy [15]. The clinician‐graded tool demonstrated good interrater reliability (91.0% agreement, Kappa = 0.81, *p* < 0.001) for the presence of LEF types. Intrarater reliability was excellent (96.1% agreement for type; 91.4% agreement for grade). Concurrent validity was supported by associations with grayscale ultrasound examination findings, which demonstrated characteristic patterns for different types of LEF, particularly in the cheek, submental, and neck regions [15].

#### 3.2.2 Courage Kenny‐Fibrosis Grading Scale

Gilchrist et al. developed and validated the Courage Kenny‐Fibrosis Grading Scale for cancer treatment‐related fibrosis in 41 cancer survivors, including 15 post‐HNC patients [31]. Face validity was established through an iterative survey process involving 43 clinicians and researchers. The scale demonstrated adequate inter‐rater reliability (ICC = 0.810) and excellent intra‐rater reliability (ICC = 0.941). Concurrent validity was supported by moderate significant associations with Common Terminology Criteria for Adverse Events (CTCAE) and LENT‐SOMA scales (rS = 0.290–0.574, *p* < 0.05) [31].

### 3.3 Imaging‐Based Tools

#### 3.3.1 Ultrasound‐Based Tools

Young's Modulus (Ultrasound Indentation): Leung et al. assessed 105 patients who had received RT to the entire neck using an ultrasound probe and computer algorithm that quantified tissue indentation due to applied force [29]. Young's modulus correlated positively with hand palpation scores and negatively with neck rotation range. Values were higher on the side receiving a radiation boost, even when palpation scores were similar, suggesting the ability to distinguish differential radiation dose exposure [29].

Zheng et al. conducted a feasibility study in 4 patients and 8 controls, reporting differences between normal tissue (12.8 ± 3.9 kPa) and fibrotic tissue (46.4–108.3 kPa) stiffness [32]. Reliability assessment showed site‐dependence variation of ±14.2%, inter‐observer variation of ±15.2%, and intra‐observer variation of ±7.2% [32]. Huang et al. extended this work by assessing quasi‐linear viscoelastic properties in 105 patients with different degrees of RIF [33].

Shear Wave Elastography (SWE): Liu et al. evaluated SWE in 50 subjects (25 post‐irradiated patients and 25 age/sex‐matched controls) [22]. The technique demonstrated excellent intraoperator reliability (ICC = 0.90–0.99) and good interoperator reliability (ICC = 0.77–0.94). SWE stiffness measurements were significantly higher in post‐irradiated patients compared to controls. SWE stiffness increased with post‐irradiation duration and correlated with muscle atrophy and subcutaneous tissue thinning [22].

Khodadadi et al. evaluated both SWE and B‐mode sequential image processing for predicting radiotherapy‐induced sternocleidomastoid muscle fibrosis in 20 sternocleidomastoid muscles, assessing patients before, during (weeks 3 and 6), and 3 months after radiotherapy [28]. SWE did not detect significant changes during radiotherapy. In contrast, B‐mode sequential image processing demonstrated that both Young's modulus and shear modulus increased significantly with cumulative radiation dose (*p* = 0.001). At 48 Gy, Young's modulus showed 100% specificity and 85% sensitivity, while shear modulus showed 95% specificity and 75% sensitivity. Both parameters also increased significantly 3 months post‐radiotherapy compared to pre‐radiotherapy (Young's modulus *p* = 0.029; shear modulus *p* = 0.004), though discriminant sensitivity dropped to 56%–63% at this timepoint. The authors introduced these B‐mode‐derived parameters as biological markers for detecting fibrosis onset during initial radiotherapy fractions [28].

Ultrasonic Nakagami Imaging: Yang et al. investigated Nakagami imaging in 40 participants classified into control (*n* = 12), asymptomatic post‐RT (*n* = 11), and symptomatic post‐RT with fibrosis (*n* = 17) groups [30]. Significant differences in all Nakagami parameters were observed between control and post‐RT groups. Compared with controls, the Nakagami shape parameter increased by 74.1% (*p* < 0.001) and scaling parameter increased by 83.5% (*p* < 0.001) in the symptomatic group [30]. Discrepancies were noted between clinician‐based and patient‐based assessments of RIF, with clinician assessments frequently underestimating fibrosis severity [30].

High‐Frequency Ultrasound: Huang et al. assessed skin fibrosis using high‐frequency ultrasound in 38 patients and 20 controls [34]. Significantly different skin thickness and integrated attenuation were observed between patients and controls, but no formal reliability assessment was reported [34].

#### 3.3.2 Magnetic Resonance Imaging (MRI)‐Based Tools

Mao et al. explored T1‐ and T2‐weighted MRI signal intensity as biomarkers for HN‐LEF in a post hoc analysis of a prospective trial [35]. T2 signal intensity differentiated edematous states, and MRI structural volumes demonstrated a strong negative correlation (*p* < 0.05) with a novel 10‐point HN‐LEF score at adjacent palpable sites [35].

#### 3.3.3 Computed Tomography (CT)‐Based Tools

West et al. evaluated the inter‐observer reliability and diagnostic accuracy of the CT Lymphedema and Fibrosis Assessment Tool (CT‐LEFAT) in 26 HNC patients assessed by five physician raters [16]. The tool showed limited performance, with inter‐rater reliability indicating only slight to fair agreement (kappa = 0.04–0.36) and intra‐observer agreement that was fair to moderate (overall kappa = 0.44). Receiver Operating Characteristic Area Under the Curve (AUC) analysis showed limited diagnostic accuracy (AUC = 0.60–0.70) [16].

### 3.4 Risk of Bias

Risk of bias was assessed using the COSMIN Risk of Bias checklist (Figure 2 and Table S2). All PROMs and clinician‐reported outcome measure studies were rated as “adequate” for methodological quality across relevant measurement properties. Imaging tool studies were more frequently rated as “inadequate” or “doubtful.”

**FIGURE 2:**
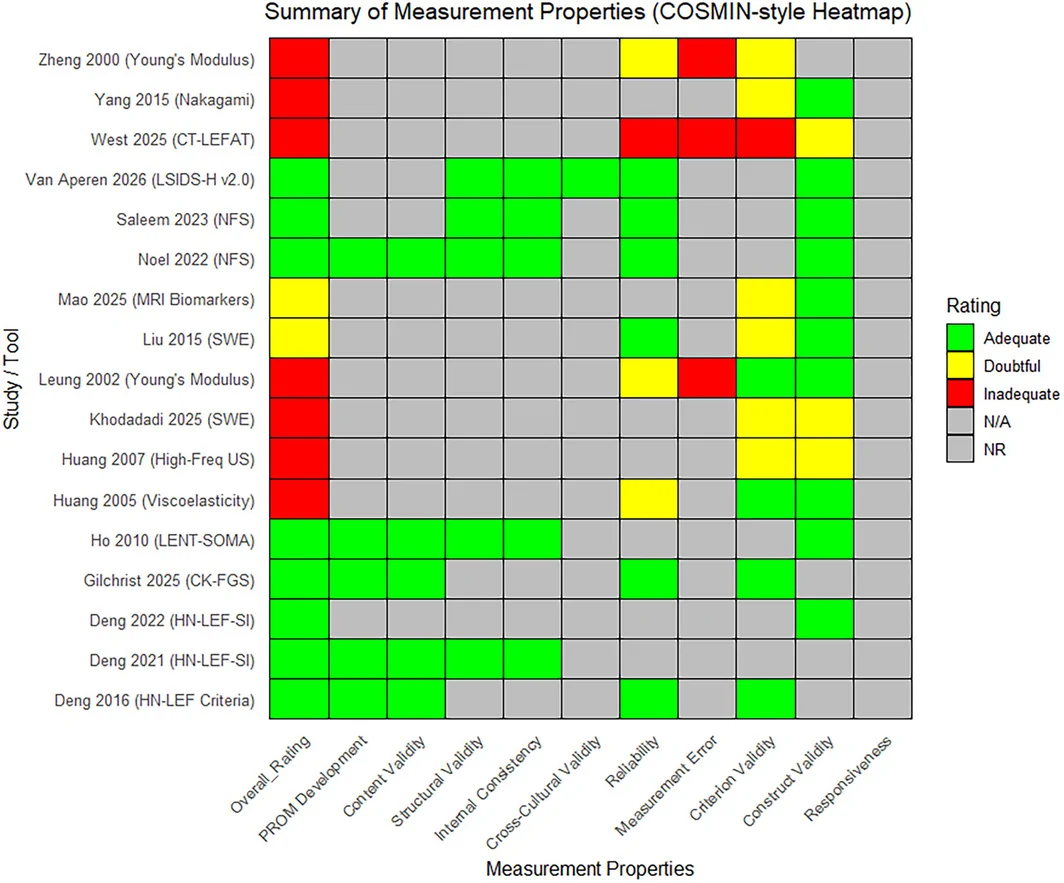
COSMIN‐based heatmap of measurement properties for head and neck radiation‐induced Fibrosis assessment tools. Each row represents a study and the tool evaluated, and each column represents a measurement property as defined by the COSMIN framework. [Color figure can be viewed at wileyonlinelibrary.com]

## 4 Discussion

In the largest systematic review of measurement tools for head and neck RIF, we identified 17 studies describing 13 distinct measurement tools for RIF in HNC patients, including 4 PROMs tools, 2 clinician‐reported outcome measures tools, and 7 imaging‐based tools.

Our findings substantially build on Shaw et al. from a decade ago [12], who identified nine studies, representing nine different tools, with only a single tool assessed for both reliability and validity. The current review suggests incremental development in PROMs with improved psychometric properties. However, the fundamental conclusion remains unchanged: there is still no consensus on a single valid, reliable, non‐invasive RIF measurement tool for use in HNC patients.

The PROMs identified in this review demonstrated adequate risk of bias ratings and acceptable psychometric properties. The NFS demonstrated high internal consistency and test–retest reliability [25, 26], with construct validity supported by correlation with neck elasticity and clinical indicators of fibrosis severity. The Dutch LSIDS‐H&N v2.0 demonstrated strong convergent validity and reliability, representing cross‐cultural validation that may support broader applicability of fibrosis assessment tools [24].

However, PROMs have inherent limitations. They capture subjective symptom burden rather than objective tissue changes and may be influenced by patient factors such as pain tolerance, psychological state, and health literacy. Additionally, some PROMs, such as the original HN‐LEF Symptom Inventory, were considered burdensome by more than 50% of participants due to length, leading to refinement into shorter versions [17].

An important observation across multiple studies was the discrepancy between clinician‐based and patient‐based assessments of RIF. Yang et al. demonstrated that clinician‐based assessments using the RT Oncology Group late morbidity scoring scheme underestimated fibrosis severity compared to patient‐reported symptoms [30]. Such discordance may influence symptom recognition and management. These findings suggest potential value in incorporating both subjective and objective measures in a comprehensive fibrosis assessment.

While imaging modalities offer the potential for objective, quantitative fibrosis assessment, our review identified several limitations. All imaging tools were rated as having an inadequate or doubtful risk of bias, primarily due to methodological shortcomings in study design and lack of formal reliability assessment. The CT‐LEFAT, despite being developed to standardize lymphedema and fibrosis diagnosis, demonstrated only slight to fair inter‐rater reliability [16]. West et al. indicated that additional materials, such as further training, refinement of imaging methods, or other processes, may be required before achieving clinically ready diagnostic performance [16].

Ultrasound‐based techniques, including Young's Modulus and SWE, demonstrate potential for quantitative assessment. Young's Modulus demonstrated correlation with hand palpation scores and neck rotation range and was described as offering a more quantitative and region‐specific assessment, with potential to distinguish areas exposed to differing radiation dose levels compared with palpation [29]. However, these techniques require specialized equipment expertise, limiting widespread clinical adoption. MRI‐based biomarkers are emerging but currently lack formal reliability data and face challenges including cost and limited availability [35].

Several factors may limit the implementation of objective imaging tools for fibrosis assessment in clinical practice. These include equipment cost, limited availability, need for specialized training, and lack of standardized acquisition protocols. Unlike hepatic elastography, where standardized multi‐institutional imaging‐based trials have established clear clinical utility [36], head and neck fibrosis assessment lacks such consensus guidelines [37]. The National Comprehensive Cancer Network Head and Neck Cancer Guidelines acknowledge that post‐treatment surveillance may be challenging due to altered anatomy and fibrosis from surgery, radiation, and/or chemotherapy; however, they do not offer specific guidance regarding tools for measuring fibrosis [37].

A notable finding of this review is the predominance of tools assessing external fibrosis, with no tool exclusively designed to measure internal fibrosis. This imbalance may be clinically relevant because internal fibrosis, affecting the pharyngeal, laryngeal, lingual, and deep muscular structures, can contribute to functional impairments including dysphagia, trismus, and airway compromise, which are among the more debilitating long‐term consequences of head and neck radiotherapy [38, 39]. The limited assessment of internal fibrosis may reflect both the accessibility of superficial tissues to palpation and ultrasound‐based techniques and the greater technical challenges associated with quantifying deep tissue changes [12]. Cross‐sectional imaging modalities such as CT and MRI offer the potential to assess internal fibrosis, and the CT‐LEFAT and quantitative MRI biomarkers identified in this review represent early efforts in this direction [16, 35]. However, both tools currently have limited reliability data. Future tool development may benefit from prioritizing the assessment of internal fibrosis, given its potential impact on functional outcomes and quality of life. Additional challenges specific to the head and neck region further impede the establishment of a gold standard. The complex anatomy of the head and neck precludes the use of some measurement modalities validated in other body regions; for example, bioimpedance techniques that have been used in limbs or limb segments cannot be easily adapted to the neck region, and the principles of ballistometry would be difficult to execute consistently given the contour of the surface anatomy [6]. Furthermore, tissue fibrosis in the neck involves several layers and produces different clinical consequences at different depths; dermal fibrosis may impair healing, subcutaneous fibrosis may confer tissue hardness, and muscle or joint fibrosis may cause restricted neck motion, trismus, and dysphagia, making it unlikely that a single measurement technique can capture all changes [6]. The coexistence of pathological processes such as tumor infiltration, lymphedema, and inflammation further complicates the isolation of fibrosis as a discrete measurable entity.

The persistently small sample sizes across studies warrant discussion given that RIF is a prevalent complication [40]. Several interrelated factors are likely to contribute. First, the heterogeneity of the HNC population, encompassing diverse subsites, each with different treatment protocols, radiation fields, and fibrosis manifestations, makes it difficult to assemble large, homogeneous cohorts for assessment studies [6]. Second, RIF typically manifests months to years after radiotherapy and can continue to progress over time, complicating prospective study design and contributing to high attrition in longitudinal studies [6, 38]. Third, the absence of a universally accepted measurement method means that studies often use novel or institution‐specific tools, limiting multi‐center collaboration and pooling of data [41]. Fourth, RIF has historically been treated as a secondary or incidental finding in oncology trials focused on tumor control and survival, rather than as a primary outcome warranting dedicated study [6, 12]. Fifth, patients with HNC face numerous competing acute and chronic toxicities, such as mucositis, xerostomia, dysphagia, and fibrosis assessment may be deprioritized in clinical follow‐up [12].

For PROM‐based or palpation‐based assessments specifically, the barrier to larger studies is not equipment cost or complexity but rather the lack of validated, standardized instruments. Clinician‐reported scales have not been rigorously tested for inter‐rater reliability or validity in the head and neck region, which discourages large‐scale adoption and multi‐site studies [41]. For imaging‐based studies, additional barriers include the cost and availability of specialized equipment, the need for trained operators, and the lack of standardized imaging protocols for fibrosis quantification in the head and neck [6]. These barriers create a circular problem: the lack of valid and reliable measurement techniques makes it difficult to properly assess the efficacy of interventions targeting RIF, which in turn reduces motivation to conduct intervention trials that would also advance assessment methodology [41].

Additionally, fibrosis is probably underreported in clinical practice because it is difficult to objectively quantify and formal methods of measurement are not well recognized, reducing the perceived clinical urgency of developing precise assessment tools [6]. The evolving treatment landscape, with the transition from conventional 2D radiotherapy to intensity‐modulated radiotherapy and proton therapy, has changed the patterns and severity of RIF, potentially rendering older studies less applicable to contemporary patient populations and complicating the establishment of standardized assessment benchmarks [42]. Furthermore, although recent research has questioned the long‐held belief that posttreatment fibrosis is irreversible, there remains no curative therapy, and patients are treated symptomatically with approaches such as pentoxifylline combined with vitamin E, hyperbaric oxygen therapy, and manual therapy [43, 44, 45]. The absence of a curative therapy may further reduce the perceived urgency of developing precise assessment tools.

This systematic review has several limitations. First, the heterogeneity of measurement tools and outcome definitions precluded meta‐analysis. Second, many studies had small sample sizes and were conducted at single institutions, limiting generalizability. Third, the COSMIN methodology, while rigorous, may not fully capture all aspects of measurement quality relevant to imaging‐based tools. Fourth, the classification of fibrosis type (internal vs. external) was performed post hoc and was based on the anatomical structures assessed in each study rather than on explicit categorization by the original study authors. Some tools, particularly PROMs that include symptom items potentially reflecting both internal and external fibrosis (e.g., tightness, difficulty swallowing), may not fit neatly into a binary classification. Finally, studies published after our updated search date may have been missed.

Our findings will help guide patient care, inform clinical trials, and support the development of novel measurement tools. For patient care, a multimodal assessment approach incorporating both patient‐reported and objective measures is essential to effectively characterize RIF. For clinical settings, the NFS may offer a validated, fibrosis‐specific PROM with favorable psychometric properties and manageable length. For objective assessment, SWE provides quantitative stiffness measurements with good intraoperator reliability, though additional research is needed to better understand its association with functional outcomes [22].

Future research may focus on head‐to‐head comparisons of existing tools to establish criterion validity, longitudinal studies assessing responsiveness to change, which is critical for monitoring treatment response, standardization of imaging protocols to improve inter‐rater reliability, development of composite assessment approaches that integrate subjective and objective measures, and validation studies in diverse populations to ensure generalizability.

Additionally, the integration of emerging technologies such as artificial intelligence‐assisted image analysis may improve the reliability and accessibility of objective fibrosis assessment. AI technologies, particularly radiomics and machine learning algorithms, can extract and quantify imaging biomarkers from CT, MRI, PET, and ultrasound that correlate with radiation‐induced tissue changes including fibrosis [46, 47]. These approaches address fundamental limitations of traditional assessment by reducing interobserver variability and operator dependency [48].

## 5 Conclusions

Despite advances in the development of RIF measurement tools for HNC patients, no single tool has emerged as the gold standard. PROMs, particularly the NFS, demonstrate adequate validity and reliability for capturing patient‐reported fibrosis burden, while imaging‐based tools offer objective quantification but require further refinement. A multimodal assessment approach incorporating both subjective and objective measures should be considered until greater consensus is established. Future research should focus on comparative validation studies and the development of standardized assessment protocols to strengthen the quality of survivorship care for HNC patients.

## Author Contributions

**Vanessa Helou:** conceptualization, data curation, investigation, methodology, writing – original draft preparation. **Pooja D. Reddy:** conceptualization, data curation, investigation, writing – review and editing. **Purva H. Rumde:** data curation, investigation, writing – review and editing. **Mary L. Klem:** conceptualization, methodology, project administration, writing – review and editing. **Sagar G. Kansara:** conceptualization, supervision, writing – review and editing. **Oldrich Virag:** data curation, investigation, writing – review and editing. **Yvonne M. Mowery:** conceptualization, supervision, writing – review and editing. **Nicole N. Scheff:** conceptualization, supervision, writing – review and editing. **Matthew E. Spector:** conceptualization, supervision, writing – review and editing. **Shaum Sridharan:** conceptualization, supervision, writing – review and editing. **Marci L. Nilsen:** conceptualization, writing – review and editing. **Kevin J. Contrera:** conceptualization, methodology, project administration, supervision, writing – review and editing.

## Funding

Dr. Contrera receives grant support from the National Institutes of Health/National Center for Advancing Translational Sciences (1 KL2 TR001856), the American College of Surgeons (Martin Faculty Research Fellowship), and the National Cancer Institute (P50CA097190 for CA230319).

## Conflicts of Interest

Dr. Contrera receives research support from Droplet Biosciences, Haystack Oncology, and Naveris not related to this work. The other authors declare no conflicts of interest.

## Supporting information


**Table S1:** Characteristics of Included Studies Assessing Radiation‐Induced Fibrosis in Head and Neck Cancer.

**Table S2:** COSMIN Risk of Bias Assessment of Head and Neck Fibrosis Measurement Tools.

## Data Availability

The data that supports the findings of this study are available in the Supporting Information of this article.

## References

[1] L. Q. M. Chow , “Head and Neck Cancer,” New England Journal of Medicine 382, no. 1 (2020): 60–72, 10.1056/NEJMra1715715. 31893516

[2] J. Sia , R. Szmyd , E. Hau , and H. E. Gee , “Molecular Mechanisms of Radiation‐Induced Cancer Cell Death: A Primer. Mini Review,” Frontiers in Cell and Development Biology 8 (2020): 2020, 10.3389/fcell.2020.00041. PMC7031160 32117972

[3] C. Chargari , E. Rassy , C. Helissey , S. Achkar , S. Francois , and E. Deutsch , “Impact of Radiation Therapy on Healthy Tissues,” International Review of Cell and Molecular Biology 376 (2023): 69–98, 10.1016/bs.ircmb.2022.11.006. 36997270

[4] I. Brook , “Late Side Effects of Radiation Treatment for Head and Neck Cancer,” Radiation Oncology Journal 38, no. 2 (2020): 84–92, 10.3857/roj.2020.00213. PMC7533405 33012151

[5] L. Kreuder , P. A. Bissey , K. W. Yip , and F. F. Liu , “Exploring Radiation‐Induced Fibrosis: Biological Mechanisms and New Frontiers in Research and Therapeutics,” International Journal of Radiation Biology (2025): 1–16, 10.1080/09553002.2025.2540353. 40892055

[6] E. C. Moloney , M. Brunner , A. J. Alexander , and J. Clark , “Quantifying Fibrosis in Head and Neck Cancer Treatment: An Overview,” Head & Neck 37, no. 8 (2015): 1225–1231, 10.1002/hed.23722. 24797251

[7] M. E. Muehlebach , S. Pradeep , X. Chen , et al., “Radiation‐Induced Fibrosis (RIF) in Head and Neck Squamous Cell Carcinoma (HNSCC): A Review,” Cells 14, no. 24 (2025): 1969, 10.3390/cells14241969. PMC12731690 41439988

[8] M. Baudelet , L. Van den Steen , P. Tomassen , et al., “Very Late Xerostomia, Dysphagia, and Neck Fibrosis After Head and Neck Radiotherapy,” Head & Neck 41, no. 10 (2019): 3594–3603, 10.1002/hed.25880. 31329343

[9] I. I. Verginadis , D. E. Citrin , B. Ky , et al., “Radiotherapy Toxicities: Mechanisms, Management, and Future Directions,” Lancet 405, no. 10475 (2025): 338–352, 10.1016/s0140-6736(24)02319-5. PMC12758832 39827884

[10] S. Shrishrimal , E. A. Kosmacek , and R. E. Oberley‐Deegan , “Reactive Oxygen Species Drive Epigenetic Changes in Radiation‐Induced Fibrosis,” Oxidative Medicine and Cellular Longevity 2019 (2019): 4278658, 10.1155/2019/4278658. PMC6381575 30881591

[11] J. M. Straub , J. New , C. D. Hamilton , C. Lominska , Y. Shnayder , and S. M. Thomas , “Radiation‐Induced Fibrosis: Mechanisms and Implications for Therapy,” Journal of Cancer Research and Clinical Oncology 141, no. 11 (2015): 1985–1994, 10.1007/s00432-015-1974-6. PMC4573901 25910988

[12] S. M. Shaw , S. A. Skoretz , B. O'Sullivan , A. Hope , L. W. Liu , and R. Martino , “Valid and Reliable Techniques for Measuring Fibrosis in Patients With Head and Neck Cancer Postradiotherapy: A Systematic Review,” Head & Neck 38, no. Suppl 1 (2016): E2322–E2334, 10.1002/hed.24249. 26559913

[13] J. Deng , E. M. Wulff‐Burchfield , and B. A. Murphy , “Late Soft Tissue Complications of Head and Neck Cancer Therapy: Lymphedema and Fibrosis,” Journal of the National Cancer Institute Monographs 2019, no. 53 (2019): lgz005, 10.1093/jncimonographs/lgz005. 31425591

[14] M. Fadhil , R. Singh , T. Havas , and I. Jacobson , “Systematic Review of Head and Neck Lymphedema Assessment,” Head & Neck 44, no. 10 (2022): 2301–2315, 10.1002/hed.27136. 35818729

[15] J. Deng , M. S. Dietrich , S. H. Ridner , A. C. Fleischer , N. Wells , and B. A. Murphy , “Preliminary Evaluation of Reliability and Validity of Head and Neck External Lymphedema and Fibrosis Assessment Criteria,” European Journal of Oncology Nursing 22 (2016): 63–70, 10.1016/j.ejon.2016.02.001. 27179894

[16] N. A. West , S. K. Attia , Z. Kaffey , et al., “Evaluating Observer Reliability and Diagnostic Accuracy of CT‐LEFAT Criteria for Post‐Treatment Head and Neck Lymphedema: A Prospective Blinded Comparative Analysis,” Oral Oncology 164 (2025): 107265, 10.1016/j.oraloncology.2025.107265. PMC12087970 40174310

[17] J. Deng , M. S. Dietrich , K. J. Niermann , et al., “Refinement and Validation of the Head and Neck Lymphedema and Fibrosis Symptom Inventory,” International Journal of Radiation Oncology, Biology, Physics 109, no. 3 (2021): 747–755, 10.1016/j.ijrobp.2020.10.003. PMC9059777 33068688

[18] M. J. Page , M. K. JE , P. M. Bossuyt , et al., “The PRISMA 2020 Statement: An Updated Guideline for Reporting Systematic Reviews,” BMJ 372 (2021): n71, 10.1136/bmj.n71. PMC8005924 33782057

[19] N. Borissov , Q. Haas , B. Minder , et al., “Reducing Systematic Review Burden Using Deduklick: A Novel, Automated, Reliable, and Explainable Deduplication Algorithm to Foster Medical Research,” Systematic Reviews 11, no. 1 (2022): 172, 10.1186/s13643-022-02045-9. PMC9382798 35978441

[20] DistillerSR , “DistillerSR Systematic Review Software, Version 2023.8.2,” accessed November 11, 2024, https://www.distillersr.com.

[21] L. B. Mokkink , E. B. M. Elsman , and C. B. Terwee , “COSMIN Guideline for Systematic Reviews of Patient‐Reported Outcome Measures Version 2.0,” Quality of Life Research 33, no. 11 (2024): 2929–2939, 10.1007/s11136-024-03761-6. PMC11541334 39198348

[22] K. H. Liu , K. Bhatia , W. Chu , L. T. He , S. F. Leung , and A. T. Ahuja , “Shear Wave Elastography—A New Quantitative Assessment of Post‐Irradiation Neck Fibrosis,” Ultraschall in der Medizin 36, no. 4 (2015): 348–354, 10.1055/s-0034-1366364. 25171602

[23] J. Deng , B. A. Murphy , K. J. Niermann , et al., “Validity Testing of the Head and Neck Lymphedema and Fibrosis Symptom Inventory,” Lymphatic Research and Biology 20, no. 6 (2022): 629–639, 10.1089/lrb.2021.0041. PMC9810345 35483066

[24] K. Van Aperen , S. Nuyts , N. Devoogdt , et al., “Cross‐Cultural Adaptation, Validity and Reliability of the Dutch Version of the Lymphedema Symptom Intensity and Distress Survey‐Head and Neck Version 2.0 (LSIDS‐H&N v2.0) in Head and Neck Cancer Patients,” Disability and Rehabilitation 48, no. 4 (2026): 1125–1140, 10.1080/09638288.2025.2554945. 40914813

[25] C. W. Noel , A. Kwinter , M. Mifsud , et al., “Quantifying Neck Fibrosis and Its Functional Implications: Development of the Neck Fibrosis Scale,” Laryngoscope 132, no. 5 (2022): 1015–1021, 10.1002/lary.29897. 34652823

[26] A. Saleem , C. W. Noel , K. Hueniken , et al., “Quantifying Neck Fibrosis: Establishing the Domain Structure of the Neck Fibrosis Scale,” Laryngoscope 133, no. 9 (2023): 2198–2202, 10.1002/lary.30497. 36479707

[27] K. F. Ho , D. J. Farnell , J. A. Routledge , et al., “Comparison of Patient‐Reported Late Treatment Toxicity (LENT‐SOMA) With Quality of Life (EORTC QLQ‐C30 and QLQ‐H&N35) Assessment After Head and Neck Radiotherapy,” Radiotherapy and Oncology 97, no. 2 (2010): 270–275, 10.1016/j.radonc.2010.01.017. 20554338

[28] A. Khodadadi , M. Mokhtari‐Dizaji , R. Ghalehtaki , and N. A. Yazdi , “Using Ultrasound Sequential Images Processing to Predict Radiotherapy‐Induced Sternocleidomastoid Muscle Fibrosis,” International Journal of Radiation Biology 101, no. 4 (2025): 411–421, 10.1080/09553002.2025.2453995. 39899279

[29] S. F. Leung , Y. Zheng , C. Y. Choi , et al., “Quantitative Measurement of Post‐Irradiation Neck Fibrosis Based on the Young Modulus: Description of a New Method and Clinical Results,” Cancer 95, no. 3 (2002): 656–662, 10.1002/cncr.10700. 12209759

[30] X. Yang , E. Yoshida , R. J. Cassidy , et al., “Quantitative Ultrasonic Nakagami Imaging of Neck Fibrosis After Head and Neck Radiation Therapy,” International Journal of Radiation Oncology, Biology, Physics 92, no. 2 (2015): 407–414, 10.1016/j.ijrobp.2015.01.006. PMC4431929 25817529

[31] L. Gilchrist , L. Franco , M. Heinen , C. Holm , and M. Weightman , “Reliability and Concurrent Validity of the Courage Kenny‐Fibrosis Grading Scale for Breast or Head and Neck Cancer‐Related Fibrosis,” Clinical Rehabilitation 40, no. 5 (2025): 603–613, 10.1177/02692155251403994. 41364489

[32] Y. P. Zheng , S. F. Leung , and A. F. Mak , “Assessment of Neck Tissue Fibrosis Using an Ultrasound Palpation System: A Feasibility Study,” Medical & Biological Engineering & Computing 38, no. 5 (2000): 497–502, 10.1007/bf02345743. 11094804

[33] Y. P. Huang , Y. P. Zheng , and S. F. Leung , “Quasi‐Linear Viscoelastic Properties of Fibrotic Neck Tissues Obtained From Ultrasound Indentation Tests in Vivo,” Clinical Biomechanics 20, no. 2 (2005): 145–154, 10.1016/j.clinbiomech.2004.09.012. 15621318

[34] Y. P. Huang , Y. P. Zheng , S. F. Leung , and A. P. Choi , “High Frequency Ultrasound Assessment of Skin Fibrosis: Clinical Results,” Ultrasound in Medicine & Biology 33, no. 8 (2007): 1191–1198, 10.1016/j.ultrasmedbio.2007.02.009. 17467155

[35] S. Mao , J. Wang , H. McMilian , et al., “Exploring Quantitative MRI Biomarkers of Head and Neck Post‐Radiation Lymphedema and Fibrosis: Post Hoc Analysis of a Prospective Trial,” Head & Neck 47, no. 5 (2025): 1487–1496, 10.1002/hed.28062. PMC12040576 39794912

[36] R. K. Sterling , A. Duarte‐Rojo , K. Patel , et al., “AASLD Practice Guideline on Imaging‐Based Noninvasive Liver Disease Assessment of Hepatic Fibrosis and Steatosis,” Hepatology 81, no. 2 (2025): 672–724, 10.1097/hep.0000000000000843. 38489518

[37] A. D. Colevas , A. J. Cmelak , D. G. Pfister , et al., “NCCN Guidelines Insights: Head and Neck Cancers, Version 2.2025,” Journal of the National Comprehensive Cancer Network 23, no. 2 (2025): 2–11, 10.6004/jnccn.2025.0007. 39938471

[38] J. P. Hyppolito , A. Hesham , G. Sunavala‐Dossabhoy , and D. D. Kim , “Fibrosis, Contractures, and Trismus: Delayed Complications of Treatment of Head and Neck Cancer,” Oral Diseases 31, no. 9 (2025): 2776–2784, 10.1111/odi.70113. 41145981

[39] A. Parsons and K. Dewan , “Dysphagia and Dysphonia After Head and Neck Cancer,” Oral Diseases 31, no. 9 (2025): 2753–2760, 10.1111/odi.15152. 39370743

[40] K. J. Taylor , C. D. Amdal , K. Bjordal , et al., “Toxicities in Long‐Term Survivors of Head and Neck Cancer‐A Multi‐National Cross‐Sectional Analysis,” International Journal of Cancer 157, no. 10 (2025): 2061–2070, 10.1002/ijc.70033. PMC12439067 40735927

[41] M. Zheng , Z. Liu , and Y. He , “Radiation‐Induced Fibrosis: Mechanisms and Therapeutic Strategies From an Immune Microenvironment Perspective,” Immunology 172, no. 4 (2024): 533–546, 10.1111/imm.13788. 38561001

[42] E. H. Pow , D. L. Kwong , A. S. McMillan , et al., “Xerostomia and Quality of Life After Intensity‐Modulated Radiotherapy vs. Conventional Radiotherapy for Early‐Stage Nasopharyngeal Carcinoma: Initial Report on a Randomized Controlled Clinical Trial,” International Journal of Radiation Oncology, Biology, Physics 66, no. 4 (2006): 981–991, 10.1016/j.ijrobp.2006.06.013. 17145528

[43] S. Delanian , R. Porcher , S. Balla‐Mekias , and J.‐L. Lefaix , “Randomized, Placebo‐Controlled Trial of Combined Pentoxifylline and Tocopherol for Regression of Superficial Radiation‐Induced Fibrosis,” Journal of Clinical Oncology 21, no. 13 (2003): 2545–2550, 10.1200/jco.2003.06.064. 12829674

[44] M. Harpsø , C. N. Andreassen , and B. V. Offersen , “Pentoxifylline and Vitamin E for Treating Radiation‐Induced Fibrosis in Breast and Head and Neck Cancer Patients,” Strahlentherapie und Onkologie 201, no. 10 (2025): 1044–1048, 10.1007/s00066-025-02423-x. 40542130

[45] H. McCillan , C. L. Warneke , S. Buoy , et al., “Manual Therapy for Fibrosis‐Related Late Effect Dysphagia in Head and Neck Cancer Survivors: The MANTLE Nonrandomized Clinical Trial,” JAMA Otolaryngology. Head & Neck Surgery 151, no. 4 (2025): 319–327, 10.1001/jamaoto.2024.5157. PMC11803512 39913160

[46] Y. Guo , E. O. Aboagye , and D. M. Gujral , “Beyond Standard Radiotherapy: An AI‐Driven Framework for Personalized Radiotherapy With a Four‐Step Classification in Head and Neck Squamous Cell Carcinoma (HNSCC) Patients,” Current Oncology Reports 27, no. 11 (2025): 1346–1357, 10.1007/s11912-025-01730-x. PMC12698768 41174258

[47] G. C. Ugwu , F. Jalali , G. Liu , G. Li , J. A. Langendijk , and B. Z. Alizadeh , “The Actual Performance of ML/AI Models in Predicting Radiation‐Induced Toxicity in Head and Neck Cancer: A Systematic Review and Meta‐Analysis,” Radiotherapy and Oncology 216 (2026): 111350, 10.1016/j.radonc.2025.111350. 41421773

[48] R. O. Alabi , M. Elmusrati , I. Leivo , A. Almangush , and A. A. Mäkitie , “Artificial Intelligence‐Driven Radiomics in Head and Neck Cancer: Current Status and Future Prospects,” International Journal of Medical Informatics 188 (2024): 105464, 10.1016/j.ijmedinf.2024.105464. 38728812

