# Supplementary material for: Measurement Tools for Radiation‐Induced Fibrosis in Head and Neck Cancer: A Systematic Review
Source: Head Neck. 2026 Jul 2;48(12):3456–68. doi: 10.1002/hed.70380 (PMC13755737; doi:10.1002/hed.70380)
Supplement: Supplementary file 1 [file HED-48-3456-s001.docx]

**Supplementary Table 2**. COSMIN Risk of Bias Assessment of Head and Neck Fibrosis Measurement Tools.

| **Study** | **PROM Development** | **Content Validity** | **Structural Validity** | **Internal Consistency** | **Cross-Cultural Validity** | **Reliability** | **Measurement Error** | **Criterion Validity** | **Construct Validity** | **Responsiveness** | **Overall Rating** |
| --- | --- | --- | --- | --- | --- | --- | --- | --- | --- | --- | --- |
| **Noel 2022^1^** | Adequate | Adequate | Adequate | Adequate | N/A | Adequate | NR | N/A | Adequate | NR | **Adequate** |
| **Saleem 2023^2^** | N/A | N/A | Adequate | Adequate | N/A | Adequate | NR | N/A | Adequate | NR | **Adequate** |
| **Deng 2021^3^** | Adequate | Adequate | Adequate | Adequate | N/A | NR | NR | N/A | NR | NR | **Adequate** |
| **Deng 2022^4^** | N/A | N/A | N/A | NR | N/A | NR | NR | N/A | Adequate | NR | **Adequate** |
| **Ho 2010^5^** | Adequate | Adequate | Adequate | Adequate | N/A | NR | NR | N/A | Adequate | NR | **Adequate** |
| **Van Aperen 2025^6^** | N/A | N/A | Adequate | Adequate | Adequate | Adequate | NR | N/A | Adequate | NR | **Adequate** |
| **Deng 2016^7^** | Adequate | Adequate | N/A | N/A | N/A | Adequate | NR | Adequate | N/A | NR | **Adequate** |
| **Gilchrist 2025^8^** | Adequate | Adequate | N/A | N/A | N/A | Adequate | NR | Adequate | N/A | NR | **Adequate** |
| **Leung 2002^9^** | N/A | N/A | N/A | N/A | N/A | Doubtful | Inadequate | Adequate | Adequate | NR | **Inadequate** |
| **Zheng 2000^10^** | N/A | N/A | N/A | N/A | N/A | Doubtful | Inadequate | Doubtful | NR | NR | **Inadequate** |
| **Huang 2005^11^** | N/A | N/A | N/A | N/A | N/A | Doubtful | NR | Adequate | Adequate | NR | **Inadequate** |
| **Liu 2015^12^** | N/A | N/A | N/A | N/A | N/A | Adequate | NR | Doubtful | Adequate | NR | **Doubtful** |
| **Khodadadi^13^ 2025** | N/A | N/A | N/A | N/A | N/A | NR | NR | Doubtful | Doubtful | NR | **Inadequate** |
| **Yang 2015^14^** | N/A | N/A | N/A | N/A | N/A | NR | NR | Doubtful | Adequate | NR | **Inadequate** |
| **Huang 2007^15^** | N/A | N/A | N/A | N/A | N/A | NR | NR | Doubtful | Doubtful | NR | **Inadequate** |
| **Mao 2025^16^** | N/A | N/A | N/A | N/A | N/A | NR | NR | Doubtful | Adequate | NR | **Doubtful** |
| **West 2025^17^** | N/A | N/A | N/A | N/A | N/A | Inadequate | Inadequate | Inadequate | Doubtful | NR | **Inadequate** |

**Abbreviations:** COSMIN, COnsensus-based Standards for the selection of health Measurement INstruments;

**Footnote:** Each row represents a study and the measurement tool evaluated. Columns correspond to COSMIN-defined measurement properties. Ratings indicate the risk of bias for each property: Adequate (low risk of bias), Doubtful (uncertain risk), Inadequate (high risk of bias), N/A (not applicable), or NR (not reported).

References

1.	Noel CW, Kwinter A, Mifsud M, et al. Quantifying Neck Fibrosis and Its Functional Implications: Development of the Neck Fibrosis Scale. *Laryngoscope*. May 2022;132(5):1015-1021. doi:10.1002/lary.29897

2.	Saleem A, Noel CW, Hueniken K, et al. Quantifying Neck Fibrosis: Establishing the Domain Structure of the Neck Fibrosis Scale. *Laryngoscope*. Sep 2023;133(9):2198-2202. doi:10.1002/lary.30497

3.	Deng J, Dietrich MS, Niermann KJ, et al. Refinement and Validation of the Head and Neck Lymphedema and Fibrosis Symptom Inventory. *Int J Radiat Oncol Biol Phys*. Mar 1 2021;109(3):747-755. doi:10.1016/j.ijrobp.2020.10.003

4.	Deng J, Murphy BA, Niermann KJ, et al. Validity Testing of the Head and Neck Lymphedema and Fibrosis Symptom Inventory. *Lymphat Res Biol*. Dec 2022;20(6):629-639. doi:10.1089/lrb.2021.0041

5.	Ho KF, Farnell DJ, Routledge JA, et al. Comparison of patient-reported late treatment toxicity (LENT-SOMA) with quality of life (EORTC QLQ-C30 and QLQ-H&N35) assessment after head and neck radiotherapy. *Radiother Oncol*. Nov 2010;97(2):270-5. doi:10.1016/j.radonc.2010.01.017

6.	Van Aperen K, Nuyts S, Devoogdt N, et al. Cross-cultural adaptation, validity and reliability of the Dutch version of the Lymphedema Symptom Intensity and Distress Survey-Head and Neck version 2.0 (LSIDS-H&N v2.0) in head and neck cancer patients. *Disabil Rehabil*. Feb 2026;48(4):1125-1140. doi:10.1080/09638288.2025.2554945

7.	Deng J, Dietrich MS, Ridner SH, Fleischer AC, Wells N, Murphy BA. Preliminary evaluation of reliability and validity of head and neck external lymphedema and fibrosis assessment criteria. *Eur J Oncol Nurs*. Jun 2016;22:63-70. doi:10.1016/j.ejon.2016.02.001

8.	Gilchrist L, Franco L, Heinen M, Holm C, Weightman M. Reliability and concurrent validity of the Courage Kenny-Fibrosis Grading Scale for breast or head and neck cancer-related fibrosis. *Clin Rehabil*. Dec 9 2025:2692155251403994. doi:10.1177/02692155251403994

9.	Leung SF, Zheng Y, Choi CY, et al. Quantitative measurement of post-irradiation neck fibrosis based on the young modulus: description of a new method and clinical results. *Cancer*. Aug 1 2002;95(3):656-62. doi:10.1002/cncr.10700

10.	Zheng YP, Leung SF, Mak AF. Assessment of neck tissue fibrosis using an ultrasound palpation system: a feasibility study. *Med Biol Eng Comput*. Sep 2000;38(5):497-502. doi:10.1007/bf02345743

11.	Huang YP, Zheng YP, Leung SF. Quasi-linear viscoelastic properties of fibrotic neck tissues obtained from ultrasound indentation tests in vivo. *Clin Biomech (Bristol)*. Feb 2005;20(2):145-54. doi:10.1016/j.clinbiomech.2004.09.012

12.	Liu KH, Bhatia K, Chu W, He LT, Leung SF, Ahuja AT. Shear Wave Elastography--A New Quantitative Assessment of Post-Irradiation Neck Fibrosis. *Ultraschall Med*. Aug 2015;36(4):348-54. doi:10.1055/s-0034-1366364

13.	Khodadadi A, Mokhtari-Dizaji M, Ghalehtaki R, Yazdi NA. Using ultrasound sequential images processing to predict radiotherapy-induced sternocleidomastoid muscle fibrosis. *Int J Radiat Biol*. 2025;101(4):411-421. doi:10.1080/09553002.2025.2453995

14.	Yang X, Yoshida E, Cassidy RJ, et al. Quantitative Ultrasonic Nakagami Imaging of Neck Fibrosis After Head and Neck Radiation Therapy. *Int J Radiat Oncol Biol Phys*. Jun 1 2015;92(2):407-14. doi:10.1016/j.ijrobp.2015.01.006

15.	Huang YP, Zheng YP, Leung SF, Choi AP. High frequency ultrasound assessment of skin fibrosis: clinical results. *Ultrasound Med Biol*. Aug 2007;33(8):1191-8. doi:10.1016/j.ultrasmedbio.2007.02.009

16.	Mao S, Wang J, McMillan H, et al. Exploring Quantitative MRI Biomarkers of Head and Neck Post-Radiation Lymphedema and Fibrosis: Post Hoc Analysis of a Prospective Trial. *Head Neck*. May 2025;47(5):1487-1496. doi:10.1002/hed.28062

17.	West NA, Attia SK, Kaffey Z, et al. Evaluating observer reliability and diagnostic accuracy of CT-LEFAT criteria for post-treatment head and neck lymphedema: A prospective blinded comparative analysis. *Oral Oncol*. May 2025;164:107265. doi:10.1016/j.oraloncology.2025.107265
