# Supplementary material for: Measurement Tools for Radiation‐Induced Fibrosis in Head and Neck Cancer: A Systematic Review
Source: Head Neck. 2026 Jul 2;48(12):3456–68. doi: 10.1002/hed.70380 (PMC13755737; doi:10.1002/hed.70380)
Supplement: Supplementary file 2 [file HED-48-3456-s002.docx]

| RIF Measurement Type | Measurement Tool | Publication | Sample Size^a^ | Age | Cancer Site | Cancer Stage | Fibrosis Location | Fibrosis Type | Treatment | Number of RT Fractions | Total Dose | Time Since Treatment Completion |
| --- | --- | --- | --- | --- | --- | --- | --- | --- | --- | --- | --- | --- |
| PROMs | Neck Fibrosis Scale (NFS) | Noel 2022^1^ | 94 | Median 59–62 yrs (range 22–88) | Oral cavity (26%), oropharynx (26%), larynx/hypopharynx (21%), nasopharynx (7%), salivary gland/parotid (7%), thyroid (7%), skin (5%) | Stage I/II: 38–51%; Stage III/IV: 49–62% | Neck | External | Surgery, RT, CRT, multimodal | NR | NR | Median 2.4–2.8 yrs (range 0.5–15) |
|  |  | Saleem 2023^2^ | 129 | Median 63 (range 22–90) | Oral cavity (38%), oropharynx (20%), larynx (15%), nasopharynx (5%), salivary gland (7%), thyroid (5%), skin (5%), others (6%) | Stage I/II: 36.0%; Stage III/IV: 54%; NR: 9% | Neck | External | Surgery, RT, CRT, multimodal | NR | NR | Median 1.6 yrs (range 0.2–20.1) |
|  | Head and Neck Lymphedema and Fibrosis Symptom Inventory | Deng 2021^3^ (development paper) | 117 | Mean 59.0 yrs (SD 10.6; range 27–82) | Oral cavity (38%) and oropharynx (62%) | Stage I/II: 31.6%; Stage III/IV: 68.4% | Head and neck | External and Internal | Surgery, RT, CRT, multimodal | NR | NR | Longitudinal: baseline, end of treatment, 3–12 months post-treatment |
|  |  | Deng 2022^4^ (validation study) | 117 | Mean 59.0 yrs (SD 10.6; range 27–82) | Oral cavity (38%) and oropharynx (62%) | Stage I/II: 31.6%; Stage III/IV: 68.4% | Head and neck | External and Internal | Surgery, RT, CRT, multimodal | NR | NR | Longitudinal: baseline, end of treatment, 3–12 months post-treatment |
|  | LENT-SOMA Patient Questionnaire | Ho 2010^5^ | 220 | Mean 59.5 years (range 28.4–82.4) | Larynx (48.6%), pharynx (29.1%), oral cavity (13.6%), salivary gland (5.0%), nasal cavity/sinus (2.7%), unknown (0.9%) | Stage I/II: 65.0%; Stage III/IV: 31.4%; NR: 3.6% | Head and neck subsites | External | Surgery, RT, CRT, multimodal | Median 16 (range 15–35) | Mean 51.1 Gy; median 50 Gy (range 40–70 Gy) | Longitudinal: pre-RT to 3 years post-RT; primary validation at 24 months |
|  | Dutch LSIDS-H v2.0 | Van Aperen 2026^6^ | 30 | Median 64.5 years (IQR 19.5) | Nasal/paranasal (16.7%), oral cavity (26.7%), oropharynx (10.0%), hypopharynx (6.7%), larynx (10.0%), salivary gland (23.3%), thyroid (3.3%), other (3.3%) | Stage I/II: 36.0%; Stage III/IV: 54%; NR: 9% | Head and neck | External and Internal | Definitive RT or CRT; postoperative RT/CRT | NR | NR | 6 weeks–12 months after RT initiation: median 24 weeks |
| Physician-Reported | HN-LEF Assessment Criteria | Deng 2016^7^ | 51 | Mean 57.1 y (SD 9.7); range 41–74 | Oropharynx (45.1%), oral cavity (19.6%), larynx (11.8%), nasopharynx (5.9%), salivary gland (5.9%), others (11.8%) | Stage I/II: 4.2%; Stage III/IV: 93.8%; NR: 2.1% | Head & neck soft tissues (peri-orbital, cheek, submental, neck, supraclavicular fossa) | External | Surgery ± chemotherapy + radiation | NR | NR | Median 12.4 months (IQR 6–25) |
|  | Courage Kenny-Fibrosis Grading Scale | Gilchrist 2025^8^ | 15 head and neck cancer survivors (of 41 total cancer survivors) | NR | Head and neck cancer (not otherwise specified) | NR | Neck, jaw, face | External | Surgery ± radiation, chemotherapy | NR | NR | Single session (60–90 min); ≥8 weeks post-treatment |
| Imaging - Ultrasound | Young's Modulus (Ultrasound Indentation) | Leung 2002^9^ | 105 | NR | Nasopharyngeal carcinoma | NR | Neck | External | Radiation therapy | NR | Biological effective dose: 110–116.9 Gy₃ (upper-middle neck) + 30–33.3 Gy₃ boost | ≥2 years |
|  |  | Zheng 2000^10^ (feasibility study) | 4 patients with post-RT neck fibrosis, 8 controls | Mean 64 ± 4 | Nasopharyngeal carcinoma | NR | Neck soft tissue | External | Radiotherapy | NR | NR | During routine clinical visits every 4-6 months |
|  |  | Huang 2005^11^ | 105 | Mean 51 ± 11 | Nasopharyngeal carcinoma (NPC) | NR | Neck | External | Radiotherapy | NR | NR | At least 2 years |
|  | Shear Wave Elastography (SWE) | Liu 2015^12^ | Reliability cohort: 30 (16 healthy, 14 NPC)  Case-control cohort: 50 (25 NPC, 25 controls) | Reliability: mean 52.6 ± 9.9 Case-control: mean 55.9 ± 7.9 | Nasopharyngeal carcinoma | NR | Subcutaneous tissue and sternocleidomastoid muscle | External | Radiotherapy ± unilateral neck dissection | NR | NR | Up to 15 years |
|  | 1. 2D-Shear Wave Elastography (Young modulus)    2. B-mode sequential image processing (Young modulus and shear modulus) | Khodadadi 2025^13^ | 20 sternocleidomastoid muscles; patient number NR | Mean age 63 ± 7 years | Larynx (60%), Tongue (30%), Lip (10%) | NR | Sternocleidomastoid muscle | External | Three-dimensional conformal radiotherapy | 30 fractions (60 Gy) in 80% of patients; 35 fractions (70 Gy) in 20% of patients | 60–70 Gy prescribed; mean SCM absorbed dose 54.54 ± 7.12 Gy | Before, during the third and sixth weeks of treatment, and 3 months after radiotherapy. |
|  | Ultrasonic Nakagami Imaging | Yang 2015^14^ | 28 post-RT patients (11 asymptomatic, 17 symptomatic), 12 controls | Asymptomatic: Mean 61.1 ± 9.6;  Symptomatic: Mean 56.8 ± 7.8 | Asymptomatic: Oropharynx/Nasopharynx (9.1%), Larynx (9.1%), Oral cavity (36.4%), Tonsil (27.3%), Salivary gland (18.2%)  Symptomatic: Oropharynx/Nasopharynx (11.8%), Larynx (17.6%), Oral cavity (52.9%), Tonsil (11.8%), Salivary gland (5.9%) | T stage Asymptomatic: T1 9.1%, T2 36.4%, T3 9.1%, T4 45.5%, Tx 0% T stage Symptomatic: T1 17.6%, T2 11.8%, T3 11.8%, T4 41.2%, Tx 17.6% | Bilateral neck | External | Intensity modulated radiation therapy ± Chemotherapy | NR | Right neck mean (Gy): Asymptomatic 59.8 ± 3.0, Symptomatic 61.1 ± 2.5  Left neck mean (Gy): Asymptomatic 59.3 ± 3.1, Symptomatic 59.2 ± 2.9 | Asymptomatic: mean 21.8 ± 7.9  Symptomatic: mean 19.7 ± 8.9 |
|  | High-Frequency Ultrasound | Huang 2007^15^ | 38 | Mean 54 (SD 11) | Nasopharyngeal carcinoma | NR | Neck (5 cm below mastoid) | External | Curative-intent RT | NR | NR | Median 3 years (range 1–19) |
| Imaging - MRI | Quantitative MRI Biomarkers | Mao 2025^16^ | 16 | Mean 68.3 (SD 7.0) | Head and neck cancer (not otherwise specified) | NR | Submental and intraoral regions | External and Internal | Curative-intent RT ± manual therapy | NR | NR | ≥2 years post-RT |
| Imaging - CT | CT-LEFAT | West 2025^17^ | 26 | NR | Oropharyngeal cancer | NR | Head and neck (anterior cervical, posterior cervical, submental, anterior neck subcutaneous fat) | External and Internal | Curative-intent RT | NR | NR | 3–6 months post-RT |

**Supplementary Table 1**. Characteristics of Included Studies Assessing Radiation-Induced Fibrosis in Head and Neck Cancer.

**Abbreviations:**

CT, computed tomography; CT-LEFAT, Computed Tomography Lymphedema and Fibrosis Assessment Tool; HNC, head and neck cancer; HN-LEF, Head and Neck Lymphedema and Fibrosis; LENT-SOMA, Late Effects Normal Tissue–Subjective, Objective, Management, Analytic; LSIDS-H&N, Lymphedema Symptom Intensity and Distress Survey–Head and Neck; MRI, magnetic resonance imaging; NFS, Neck Fibrosis Scale; NR, not reported; PROMs, patient-reported outcome measures; RT, radiation therapy; SWE, shear wave elastography; US, ultrasound.

**Footnotes:**

^a^ Sample sizes reflect the number of HNC patients included in the validation cohort. For studies including mixed cancer populations, the HNC-specific sample size is reported in parentheses. For imaging studies, sample sizes may include both patients and healthy controls; these are reported separately where applicable.

External fibrosis refers to fibrosis of the skin, subcutaneous tissues, and superficial cervical musculature. Internal fibrosis refers to fibrosis of the pharyngeal, laryngeal, lingual, or deep muscular structures within the aerodigestive tract. Tools classified as 'External and Internal' assess both superficial and deep tissue compartments.

**References**

1.	Noel CW, Kwinter A, Mifsud M, et al. Quantifying Neck Fibrosis and Its Functional Implications: Development of the Neck Fibrosis Scale. *Laryngoscope*. May 2022;132(5):1015-1021. doi:10.1002/lary.29897

2.	Saleem A, Noel CW, Hueniken K, et al. Quantifying Neck Fibrosis: Establishing the Domain Structure of the Neck Fibrosis Scale. *Laryngoscope*. Sep 2023;133(9):2198-2202. doi:10.1002/lary.30497

3.	Deng J, Dietrich MS, Niermann KJ, et al. Refinement and Validation of the Head and Neck Lymphedema and Fibrosis Symptom Inventory. *Int J Radiat Oncol Biol Phys*. Mar 1 2021;109(3):747-755. doi:10.1016/j.ijrobp.2020.10.003

4.	Deng J, Murphy BA, Niermann KJ, et al. Validity Testing of the Head and Neck Lymphedema and Fibrosis Symptom Inventory. *Lymphat Res Biol*. Dec 2022;20(6):629-639. doi:10.1089/lrb.2021.0041

5.	Ho KF, Farnell DJ, Routledge JA, et al. Comparison of patient-reported late treatment toxicity (LENT-SOMA) with quality of life (EORTC QLQ-C30 and QLQ-H&N35) assessment after head and neck radiotherapy. *Radiother Oncol*. Nov 2010;97(2):270-5. doi:10.1016/j.radonc.2010.01.017

6.	Van Aperen K, Nuyts S, Devoogdt N, et al. Cross-cultural adaptation, validity and reliability of the Dutch version of the Lymphedema Symptom Intensity and Distress Survey-Head and Neck version 2.0 (LSIDS-H&N v2.0) in head and neck cancer patients. *Disabil Rehabil*. Feb 2026;48(4):1125-1140. doi:10.1080/09638288.2025.2554945

7.	Deng J, Dietrich MS, Ridner SH, Fleischer AC, Wells N, Murphy BA. Preliminary evaluation of reliability and validity of head and neck external lymphedema and fibrosis assessment criteria. *Eur J Oncol Nurs*. Jun 2016;22:63-70. doi:10.1016/j.ejon.2016.02.001

8.	Gilchrist L, Franco L, Heinen M, Holm C, Weightman M. Reliability and concurrent validity of the Courage Kenny-Fibrosis Grading Scale for breast or head and neck cancer-related fibrosis. *Clin Rehabil*. Dec 9 2025:2692155251403994. doi:10.1177/02692155251403994

9.	Leung SF, Zheng Y, Choi CY, et al. Quantitative measurement of post-irradiation neck fibrosis based on the young modulus: description of a new method and clinical results. *Cancer*. Aug 1 2002;95(3):656-62. doi:10.1002/cncr.10700

10.	Zheng YP, Leung SF, Mak AF. Assessment of neck tissue fibrosis using an ultrasound palpation system: a feasibility study. *Med Biol Eng Comput*. Sep 2000;38(5):497-502. doi:10.1007/bf02345743

11.	Huang YP, Zheng YP, Leung SF. Quasi-linear viscoelastic properties of fibrotic neck tissues obtained from ultrasound indentation tests in vivo. *Clin Biomech (Bristol)*. Feb 2005;20(2):145-54. doi:10.1016/j.clinbiomech.2004.09.012

12.	Liu KH, Bhatia K, Chu W, He LT, Leung SF, Ahuja AT. Shear Wave Elastography--A New Quantitative Assessment of Post-Irradiation Neck Fibrosis. *Ultraschall Med*. Aug 2015;36(4):348-54. doi:10.1055/s-0034-1366364

13.	Khodadadi A, Mokhtari-Dizaji M, Ghalehtaki R, Yazdi NA. Using ultrasound sequential images processing to predict radiotherapy-induced sternocleidomastoid muscle fibrosis. *Int J Radiat Biol*. 2025;101(4):411-421. doi:10.1080/09553002.2025.2453995

14.	Yang X, Yoshida E, Cassidy RJ, et al. Quantitative Ultrasonic Nakagami Imaging of Neck Fibrosis After Head and Neck Radiation Therapy. *Int J Radiat Oncol Biol Phys*. Jun 1 2015;92(2):407-14. doi:10.1016/j.ijrobp.2015.01.006

15.	Huang YP, Zheng YP, Leung SF, Choi AP. High frequency ultrasound assessment of skin fibrosis: clinical results. *Ultrasound Med Biol*. Aug 2007;33(8):1191-8. doi:10.1016/j.ultrasmedbio.2007.02.009

16.	Mao S, Wang J, McMillan H, et al. Exploring Quantitative MRI Biomarkers of Head and Neck Post-Radiation Lymphedema and Fibrosis: Post Hoc Analysis of a Prospective Trial. *Head Neck*. May 2025;47(5):1487-1496. doi:10.1002/hed.28062

17.	West NA, Attia SK, Kaffey Z, et al. Evaluating observer reliability and diagnostic accuracy of CT-LEFAT criteria for post-treatment head and neck lymphedema: A prospective blinded comparative analysis. *Oral Oncol*. May 2025;164:107265. doi:10.1016/j.oraloncology.2025.107265
